# The Christian Orthodox Church Fasting Diet Is Associated with Lower Levels of Depression and Anxiety and a Better Cognitive Performance in Middle Life

**DOI:** 10.3390/nu13020627

**Published:** 2021-02-15

**Authors:** Cleanthe Spanaki, Nikolaos E. Rodopaios, Alexandra Koulouri, Triantafyllos Pliakas, Sousana K. Papadopoulou, Eleni Vasara, Petros Skepastianos, Tatiana Serafeim, Iro Boura, Emmanouil Dermitzakis, Anthony Kafatos

**Affiliations:** 1 Department of Neurology, School of Medicine, University of Crete, Voutes, 71003 Iraklion, Greece; boura.iro@gmail.com; 2 Department of Social Medicine, Preventive Medicine and Nutrition Clinic, School of Medicine, University of Crete, Voutes, 71003 Iraklion, Greece; nikow1966@yahoo.gr (N.E.R.); alexkolou@yahoo.com (A.K.); kafatos@med.uoc.gr (A.K.); 3 Department of Public Health, Environments and Society, Faculty of Public Health and Policy, London School of Hygiene & Tropical Medicine, 15–17 Tavistock Place, London WC1H 9SH, UK; trintafyllos.pliakas@lshtm.ac.uk; 4 Impact Epilysis, 30 Psellou Michail Street, 54655 Thessaloniki, Greece; 5 Department of Nutritional Sciences and Dietetics, Faculty of Health Sciences, International Hellenic University, 57001 Thessaloniki, Greece; souzpapa@gmail.com; 6 Laboratory of Animal Physiology, Department of Zoology, School of Biology, Aristotle University of Thessaloniki, 54124 Thessaloniki, Greece; evasara@bio.auth.gr; 7 Department of Medical Laboratory Studies, Alexander Technological and Educational Institute of Thessaloniki, Sindos, 57400 Thessaloniki, Greece; pskep@otenet.gr; 8 Psychiatric Clinic, 424 General Military Hospital, N. Efkarpia, 56429 Thessaloniki, Greece; tatiana.serafeim@gmail.com; 9 Department of Genetic Medicine and Development, Faculty of Medicine, University of Geneva, Genome Center, 1211 Geneva, Switzerland; emmanouil.Dermitzakis@unige.ch

**Keywords:** Christian Orthodox Church, fasting, depression, anxiety, cognition, nutrition, Mediterranean diet

## Abstract

Lifestyle choices significantly influence mental health in later life. In this study we investigated the effects of the Christian Orthodox Church (COC) fasting diet, which includes long-term regular abstinence from animal-based products for half the calendar year, on cognitive function and emotional wellbeing of healthy adults. Two groups of fasting and non-fasting individuals were evaluated regarding their cognitive performance and the presence of anxiety and depression using the Mini Mental Examination Scale, the Hamilton Anxiety Scale, and the Geriatric Depression Scale (GDS), respectively. Data on physical activity, smoking, and vitamin levels were collected and correlated with mental health scoring. Negative binomial regression was performed to examine differences in the GDS scores between the two groups. Significantly lower levels of anxiety (7.48 ± 4.98 vs. 9.71 ± 5.25; *p* < 0.001) and depression (2.24 ± 1.77 vs. 3.5 ± 2.52; *p* < 0.001), along with better cognitive function (29.15 ± 0.79 vs. 28.64 ± 1.27; *p* < 0.001), were noticed in fasting compared to non-fasting individuals. GDS score was 31% lower (Incidence Rate Ratio: 0.69, 95% Confidence Interval: 0.56–0.85) in the fasting group compared to the control, while vitamin and ferrum levels did not differ. The COC fasting diet was found to have an independent positive impact on cognition and mood in middle-aged and elderly individuals.

## 1. Introduction

Informing lifestyle choices to promote mentally healthy aging is an important public health goal [1,2]. The population of adults over the age of 65 is projected to reach 2.37 billion by 2100 [3]. Therefore, implementation of public health strategies that promote mental and emotional wellbeing in the aging population would support the sustainable development of healthcare systems [4]. In this context, numerous studies have highlighted beneficial or detrimental dietary patterns, micro- and macronutrients, and nutritional combinations [5,6,7,8,9]. Specifically, the Mediterranean diet has been widely described as an ecological and viable nutritional model [10,11] which is associated with reduced mortality along with a lower risk for cancer, metabolic syndrome and diabetes, cardiovascular events, strokes [12,13], cognitive impairment [14,15,16], and depression in selected populations [13,17]. Adherence to the Mediterranean diet is associated with lower levels of depression, better cognitive performance, and lower dementia rates in the Greek elderly population [13]. The Mediterranean diet includes a large amount of plant foods such as vegetables, legumes, cereals, nuts, and seeds, moderate amount of dairy products, and low amount of fish, meat, and wine, while olive oil is the main source of fat [10,11]. It is enriched in beneficial nutrients that are important for brain function such as monounsaturated fatty acids, a balanced ratio of (n-6):(n-3) essential fatty acids, vitamins E and C, resveratrol, polyphenols, selenium, and glutathione, and it contains high amounts of fibers [10,11]. The mechanisms that mediate its benefits on mental health involve the anti-inflammatory and antioxidant effects of its components [18] that are exerted directly or through gut microbiota and brain-gut communication. Recent evidence suggests that the pronounced impact of the Mediterranean diet on mental health probably extends beyond the individual effects of nutrients and relates to nutrients and lifestyle behavior combinations, interactions, and cumulative effect. A new field named “nutritional psychiatry” has emerged and aims to study the relationship between dietary factors and neuropsychiatric disorders and to identify nutritional “therapeutic” interventions that can improve the course of mental diseases [19].

The Christian Orthodox Church (COC) fasting diet is widely incorporated in the Mediterranean diet [20,21]. It follows a restrictive dietary pattern with regularly repeated periods of abstinence from animal-based products and increased consumption of fruit, vegetables, and legumes, covering half a calendar year (supplementary Table S1). The COC fasting diet has been associated with low levels of saturated fatty acids and high levels of fibers and folate during the fasting periods [20]. Faithful Christians strictly adhere to this restrictive diet since childhood and throughout their lifespan. However, data on the effect of these long-lasting intermittent dietary restrictions on the mental health of people post-middle age are lacking.

In the present study, we have investigated the impact of the COC fasting diet on specific measurements of depression, anxiety, and cognitive function of healthy middle-aged and elderly individuals.

## 2. Materials and Methods

### 2.1. Subjects

Subjects were randomly recruited from the broader area of Thessaloniki, the largest city in northern Greece, including churches, monasteries, and various workplaces. Two groups were studied. The first group included people who vigorously adhered to all COC fasting practices during all recommended periods since childhood. The second group included individuals who were not vegetarian and had never fasted. A history of chronic, systemic, neurodegenerative, or psychiatric diseases and any current medical treatment or dietary supplementation on a regular basis constituted exclusion criterion for both groups. All participants signed an informed consent which was approved by the Ethics Committee of the University of Crete.

### 2.2. Study Design

Epidemiological data on gender, age, educational level, residency, and smoking were collected. Past medical history, comorbidities, current and previous medications, as well as anthropometric measures such as weight, body mass index (BMI), coded as normal, overweight, and obese individuals, body fat distribution, as measured by Dual-Energy X-ray Absorption (DEXA), waist circumference, and waist-to-hip ratio, were recorded. History of any recent weight changes was obtained and used as an indicator of current general health status. Data on the type, frequency (times per week), and duration (hours per week) of any physical activity were also collected and coded as 0 to reflect absence of any physical activity or activity practiced only rarely (less than one hour per week), and 1 to reflect moderate to vigorous physical activity (one or more hours per week). Finally, levels of vitamin B12, vitamin D, folate, and ferrum were measured and treated as secondary outcomes.

### 2.3. Dietary Data

Dietary data and alcohol intake were assessed as previously described [22]. Specifically, data were collected through interview-based recalls of food intake over three days, which included a Wednesday or Friday (during which the fasters obeyed fasting), another weekday, and a weekend day. Participants were asked to report all foods and liquids consumed during those days. Food intake records were analyzed using the Food Processor Nutrition Analysis software (ESHA, Salem, OR). In addition to the food intake records, a semi-quantitative food frequency questionnaire with 140 questions was used to assess the participants’ dietary habits during a period of one month. Frequency of consumption was based on a typical portion of each food and beverage.

### 2.4. Mental Health Evaluation

The primary outcome of the study was the evaluation of the cognitive performance and the presence of depression and anxiety in all individuals as well as the comparison of these parameters between the fasting and non-fasting group. To achieve this, we used three widely used clinical scales, those being the Mini Mental State Examination Scale (MMSE) [23], the 15-item Geriatric Depression Scale (GDS) [24], and the Hamilton Anxiety Scale (HAS) [25].

### 2.5. Statistical Analysis

Summary statistics were produced for each group (fasting vs. control group). Categorical variables were expressed as frequencies with percentages. Continuous variables were expressed as means with standard deviation. All primary and secondary outcomes were skewed, and the non-parametric two-sample Wilcoxon rank-sum (Mann-Whitney) test was used to assess differences in mental health indicators and micronutrients between the fasting and non-fasting group. As depressive symptoms have been consistently associated with worse cognitive performances, a correlation analysis was performed between GDS and HAS with MMSE scores in the two groups. Correlation analysis was also performed with the primary outcomes and B12 levels. We also examined the GDS score in regression analysis. Specifically, a negative binomial regression was used to examine differences in the two groups treating the score as a count variable. Four models were produced. The first model provided unadjusted estimates. The second model was adjusted for the socio-demographic variables (age, gender, education). The third model was further adjusted for body fatness, general health status, smoking, and physical activity, and the final model was also adjusted for monk status. In sensitivity analysis, we restricted the analysis to those having a GDS score above the 75th percentile (GDS score >3, n = 20 in the fasting and n = 49 in the control group).

## 3. Results

**Figure 1:**
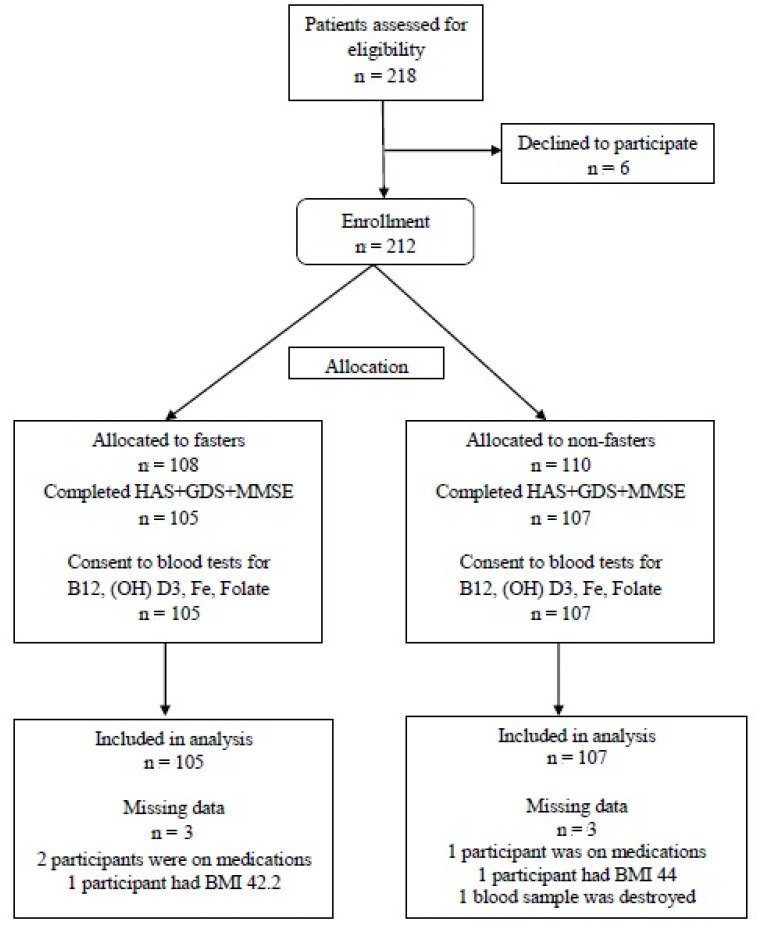
Participants Flow Chart.

A total of 218 healthy individuals were recruited from September 2013 to October 2015, from whom six were excluded (three due to concomitant medications, two due to high BMI, and one due to blood drawing refusal). Finally, 105 subjects were allocated to the fasting group and 107 to the non-fasting (control) group. A flow chart of all participants is presented in Figure 1.

### 3.1. Sociodemographic, Health, and Diet Characteristics

Participants in the fasting and non-fasting groups did not significantly differ in the distribution of gender, age, education level, BMI, and recent weight changes, with the latter suggesting a similar general health status. The fasting group included significantly fewer smokers than the non-fasting group (*p* < 0.001) and nine monks compared to none in the non-fasting group (*p* = 0.002). Types of physical activity were comparable in the two groups and included mainly walking, cycling, dancing, swimming, and aerobic exercise. Individuals who reported a regular moderate-to-vigorous activity were equally distributed between the two groups. No participants of either group reported attending a structured physical exercise program. Summary statistics for the two groups (fasting vs. non-fasting) are presented in Table 1.

Dietary habits of the two groups derived from the 30-day dietary recall. Fasters and controls differed significantly in several food groups with fasters consuming more frequently seafood, fruits, cereals, and olive oil, whereas non-fasters meat and dairy products, as well as alcohol and coffee. Specifically, a higher percentage of non-fasters (29%) reported regular alcohol consumption compared to fasters (14%) (*p* = 0.002). Detailed comparisons of the drinking and dietary habits in the two groups are presented in the supplementary Tables S2 and S3.

### 3.2. Vitamins B12, 25 (OH) D3, Folate, and Ferrum

Participants in the non-fasting group had significantly lower levels of folate compared to the fasting group (*p* = 0.005). Nevertheless, both groups showed similarly low levels of vitamin 25 (OH) D3 and normal levels of vitamin B12 and ferrum. Summary statistics for the two groups are presented in Table 2.

### 3.3. Depression and Anxiety

Levels of depression and anxiety, as well as cognitive performance scores, are presented in Table 3. Individuals in the fasting group had significantly lower scores in GDS (*p* < 0.001) and HAS (*p* < 0.001). These results were also applicable when examining males and females separately.

Negative binomial regression was used for the GDS score, revealing a statistically significant difference between the two groups. As depicted in Table 4, the GDS score was found 36% (IRR: 0.64, 95% CIs: 0.52–0.79) lower in the fasting group compared to the control group in the unadjusted model and 31% (IRR: 0.69, 95% CIs: 0.56–0.85) in the fully adjusted model.

In sensitivity analysis we found that, in the fully adjusted model, the GDS score was 8% lower in the fasting group (IRR: 0.92, 95% CIs: 0.77–1.10), although this difference was not statistically significant (Table 5).

In total, 26 individuals reported symptoms of mild depression in the GDS evaluation, six being fasters and 20 non-fasters (5.7% and 18.7%, respectively in their groups), with this difference being statistically significant (*p* = 0.004). No individuals with moderate or severe depression were found in either group. Using the HAS, we identified 17 individuals in our sample that reported symptoms of anxiety, six being fasters and 11 non-fasters. All six fasters reported only symptoms of mild anxiety, whereas seven non-fasters reported mild to moderate anxiety symptoms and four moderate to severe (Table 6).

**Figure 2:**
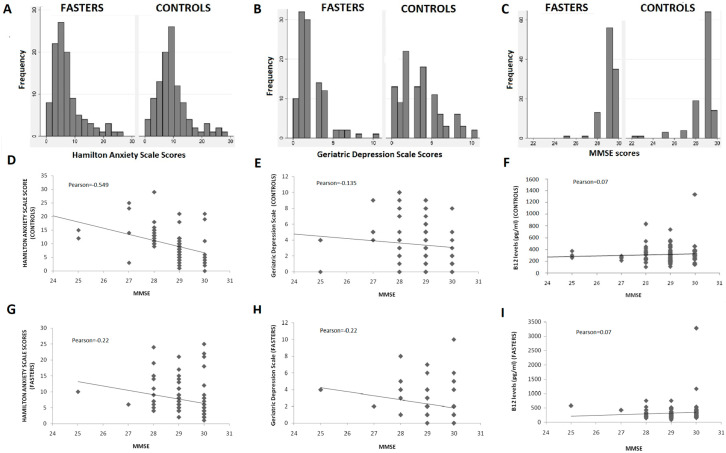
(**A**–**C**): Distribution of GDS, HAS, and MMSE scores; (**D**–**I**): Correlation of GDS, HAS, and B12 levels with MMSE scores.

Detailed comparison of the GDS and HAS scores distribution in the two study groups (Figure 2A,B) further support a protective effect of COC fasting diet on depression and anxiety.

The majority of fasters reported no symptoms suggestive of depression (scores < 3 in GDS) or anxiety (scores < 6 in the HAS). On the other hand, almost half of non-fasters reported scores > 3 and > 6 in the GDS and HAS, respectively (Figure 2A,B). Furthermore, individuals with clinically meaningful symptoms of anxiety and depression were found to be more prevalent in the non-fasting group as compared to the fasting one. The difference was statistically significant for the GDS scores but did not reach statistical significance in the HAS dataset.

### 3.4. Cognitive Performance

Fasting individuals had significantly higher scores in the MMSE as compared to non-fasters with this difference being statistically significant (*p* < 0.001) (Table 3). Although the overall distribution of scores was comparable in the two groups (Figure 2C), individuals with MMSE < 27 were more prevalent among non-fasters than among fasters, while two individuals with MMSE of 22 were identified in the non-fasting group. No individual was found to have a clinically significant decrease of MMSE (<24) in the fasting group (Figure 2C).

MMSE scores were correlated with GDS and HAS scores in the two groups with results being presented in Figure 2D,E,G,H. No significant differences were detected. Correlation between symptoms of depression or anxiety and cognitive performance was almost identical in the two groups, except for a negative correlation found between anxiety and cognitive performance, which was stronger in the control group (Pearson: −0.549) compared to the fasting (Pearson: −0.22).

Vitamin B12 levels did not differ between the two groups, although they fluctuated more among fasters (mean = 330 ± 327 pg/mL) compared to controls (316 ± 155 pg/mL). B12 levels were not found to correlate significantly with MMSE scores in either group (Figure 2F,I) and the same was true for GDS or HAS scores (data not shown).

## 4. Discussion

In the present study, 105 faithful fasters and 107 non-fasting individuals were studied comparatively regarding their performance on three well-known and commonly used scales, which evaluate cognition and the presence of anxiety or depression. Our results revealed that fasters achieved better scores in all three scales compared to the non-fasting participants. Despite the differences noticed between the two groups, scores obtained were largely within the normal range. The differences between the two groups in depression scores reached statistical significance, even when adjusted for socio-demographic and anthropometric data. Hence, the present study provides evidence supporting a beneficial effect of the COC fasting diet on the psychological and mental health of middle-aged and elderly adults.

The COC fasting rituals involve a periodic alternating pattern between omnivore and vegetarian diet that allows seafood consumption and occasionally fish. It is applied for 180–200 days that are periodically scattered throughout the year. Additionally, total abstinence from olive oil, meat, fish, milk, and dairy products is recommended every Wednesday and Friday throughout the year. There are three fasting periods: preceding Christmas (40 days) and Easter (Lent, 48 days) and 15 days in August (the Assumption) when meat, dairy products, and eggs are not allowed. While fish and olive oil are permitted during the first fasting period (except for Wednesdays and Fridays), during Lent fish can only be consumed on weekends. During the Assumption, fish is permitted only on August 6th. Seafood such as shrimps, squid, cuttlefish, octopus, lobsters, crabs, and snails are allowed throughout the year (supplementary Table S1).

This religious diet is reported to have important health-promoting characteristics. It is enriched in micronutrients and single food items or groups that have been associated with a better mental health status. Specifically, it involves low intake of dietary cholesterol, total and saturated fat, and trans-fatty acids, and high intake of antioxidant vitamins, dietary fibers, folate, and carbohydrates during fasting versus non-fasting periods [22,26,27,28,29] while ferrum levels are not significantly affected by fasting [30]. Previous studies have shown that increased consumption of fruits, vegetables, fish, and seafood was associated with a decreased risk for depression and cognitive impairment and lower levels of anxiety [31,32,33,34]. In our study, fasters consumed less meat, dairy products, olive oil, and processed, sugar-containing food and beverages, and more nuts, fresh seasonal fruits, seafood, and healthy plant-derived fats such as tahini, compared to non-fasters. These findings suggest that fasters adopt early in their life healthier dietary habits in the context of this religious diet, a factor that could account for their better performance in mental health measurements. On the other hand, while physical activity was similarly practiced between the two groups, smoking, alcohol, and coffee consumption were significantly more prevalent among non-fasters compared to fasters. It is widely accepted that smoking and alcohol are associated with both anxiety and depression in a two-way relation and exert a detrimental effect on brain function. Considering the above, it may be possible that the differences in mental health scoring observed between the two groups in our study may reflect a combination of both the beneficial effect of fasters’ early adoption of a healthier diet and the detrimental effect on brain function of the non-fasters’ Western-type diet that includes regular toxic substances consumption.

Although the word “fasting” literally means complete food deprivation, the COC religious diet does not involve days of total food abstinence. Instead, it requires a periodic pattern of abstinence from energy-rich, mainly animal-based products that is adopted at a noticeably young age, usually during childhood, and is maintained with high adherence until old age (supplementary Table S1). A recent study in Athonian monks reported a decrease of 400 kcal in daily energy intake during fasting periods [35]. This reduction was even larger in faithful fasters of the general population, reaching 25% of the total caloric intake of non-fasting days (about 570 kcal) [36]. Based on this evidence, the COC religious diet can be considered a periodic intermittent fasting diet. Intermittent fasting is defined as the dietary pattern that includes extended time periods (hours or days) with little or no energy intake, alternating with periods of normal food intake. The term periodic fasting is used to describe the type of intermittent fasting that includes periods with either complete abstinence from food or food restriction lasting several days [37]. Based on this definition, the COC fasting diet is a type of periodic diet with regularly repeated food restriction periods of various durations, covering 1 (Wednesdays and Fridays) to 41 days (Lent) (supplementary Table S1). Several studies have suggested that intermittent versus continuous energy restriction is associated with greater improvement in several health indicators, such as lipid profile, insulin resistance, leptin sensitivity, glucose homeostasis, blood pressure, inflammation, resistance to stress, and so forth [37,38,39,40]. Animal studies have revealed that intermittent fasting can improve the course of several diseases such as cancer, metabolic syndrome, or cerebrovascular diseases, while they can delay the onset or slow the progression of common neurodegenerative disorders, like Alzheimer’s and Parkinson’s disease [40]. The underlying mechanisms of intermittent diet’s beneficial effects include activation of adaptive cellular stress response, enhancement of mitochondrial health, DNA repair, and autophagy [37,38]. While the COC fasting diet results in moderate energy restriction during fasting periods (25%), the long durations of these fasting periods, its adoption at a young age, and its maintenance for decades until advanced age may play a role in its beneficial effects. Several studies suggest that when healthy diets are adopted early and maintained for long, they can have more pronounced effect in health improvement. Based on the above, it is not surprising that we have found improved mood and cognition in individuals that fasted all their life compared to those who never did.

The COC fasting diet is considered an important “hidden” element of the Mediterranean diet [20,30] that could account, at least partly, for its beneficial effects. In fact, the favorable effect of the “Mediterranean diet” on Cretans’ survival and health status during the Seven Countries Study [30] could have been attributed to the widespread practice of COC fasting rituals, a factor that was largely ignored by the study researchers.

Several factors make our study unique. Firstly, all participants were carefully selected to represent healthy middle-aged and elderly individuals, with no known, suspected, or previously reported diseases, who took no medications or supplements. Hence, any confounding factor deriving from chronic disease burden that could have affected mood and cognition was eliminated. Secondly, the adherence of faithful Christians to this periodic religious diet is the highest possible as their self-discipline is internally motivated. Finally, in our sample of fasting individuals, the adherence to the COC fasting rituals started during early childhood and was maintained without interruption to old age. Such a high and long-lasting adherence to a diet could never be achieved in predesigned double-blind nutritional case-control studies. Thus, our sample provided a valuable opportunity to investigate the long-term effects of long-lasting intermittent abstinence from animal-based food in real life.

Our results gain further interest in the light of the increasingly reported psychiatric symptoms in many countries’ populations, including Greece. According to the European Core Health Indicator, the prevalence of self-reported depression in Greece rose from 2.3% in 2009 to 4.7% in 2014 [40], while suicide rates have reached a peak in recent years in the context of the economic crisis [41]. Although major depression is less common in older adults compared to the younger, it may exert a detrimental impact on multiple aspects of their life, including cognitive performance [42], while its prevalence is expected to rise soon [43]. What is more, in older adults, subthreshold depression was encountered twice or three times more often than major depression with almost 1 out of 10 of these individuals deteriorating to major depression per year [44]. Towards this direction, in our study the prevalence of subthreshold depression was threefold higher (17.8%) among healthy middle-aged and old non-fasting individuals as compared to fasting individuals (5.6%) of similar demographic characteristics. Despite the magnitude of the problem, evidence-based public health strategies to prevent anxiety and depression in this age spectrum are limited [45,46,47].

The present study was not without limitations. Fasters are, by definition, deeply religious people who tend to adopt special habits and social behaviors, including monasticism, finding joy in helping others, spiritual fellowships, and attending regularly places of worship. The significant spiritual effect of this religiosity on mood, self-esteem, and life satisfaction cannot be ignored [48,49,50]. Furthermore, religious fasting should be considered as an ascetic approach of self-discipline in the context of faith and spiritual reward, accompanied by increased mental alertness and a deep sense of internal peace that can result in improved mental health [51]. This approach might have introduced bias in our study, for which our data could not be adjusted. On the other hand, spirituality and diet have a bidirectional relationship. A diet with anti-inflammatory and antioxidant properties can improve brain function, enhance perception, and promote spirituality. An additional important confounding factor is the healthier lifestyle which is usually adopted by fasters compared to non-fasters. Rates of mental illness are, indeed, higher among smokers, although this tendency is more profound in younger individuals [52] and those with concomitant anxiety [53]. Smoking and alcohol consumption has been recently recognized as a causal risk factor for several psychiatric conditions [54]. In our study, both smoking and alcohol were more prevalent among non-fasters and, although adjustment for smoking did not affect our results on depression, a smoking- or alcohol-associated detrimental effect on cognition or a coexistence of smoking and alcohol consumption with anxiety and depression cannot be excluded [55]. A synergistic effect of healthy or unhealthy dietary habits and specific lifestyle behaviors on mental health is possible.

Furthermore, our participants have not undergone a complete neuropsychological evaluation, and detection of depression, anxiety, and cognitive decline through scales is not always reliable. Finally, it can be argued that, although differences between fasters and non-fasters in mental health evaluation were “statistically significant”, absolute scoring values were within normal range and as such, they may not be “clinically significant”. However, on an individual basis, even a single point on any of these scales, despite being within the range of “normality”, could have a great impact on the everyday quality of life and as such, it does matter. Our epidemiological study does not provide evidence about causality between the COC fasting diet and the improved mental health status of fasting individuals. Future studies should be designed to reproduce our finding in larger cohorts and investigate the underlying mechanisms of this association.

Considering the above, we do not suggest the COC fasting diet as a therapeutic intervention for psychological and mental health disorders. However, we do believe it could be encouraged as a sustainable way of nourishment, especially for populations of a COC religious background that long to maintain mental sanity and psychological balance throughout the human lifespan.

## Figures and Tables

**Table 1:** Demographic and anthropometric characteristics of fasting and non-fasting individuals.

	Fasting Group (n = 105) n (%)		Control Group (n = 107) n (%)		*p*-Value ^1^
**Gender**					
Males	37 (35.2%)		33 (30.8%)		0.496
Females	68 (64.8%)		74 (69.2%)		
**Education**					
Primary	9 (8.6%)		20 (18.7%)		
Secondary	39 (37.1%)		41 (38.3%)		
Tertiary	44 (41.9%)		39 (36.4%)		0.098
Postgraduate	13 (12.4%)		7 (6.5%)		
**Recent weight change**					0.808
Yes	20 (19.0%)		19 (17.8%)		
No	85 (81.0%)		88 (82.2%)		
**Smoking**	4 (3.8%)		39 (36.4%)		**<0.001**
**Alcohol**					**0.002**
No	91 (86.7%)		78 (72.9%)		
Yes	14 (13.3%)		29 (27.1%)		
**Physical activity ^2^**					0.352
No	74 (70.5%)		69 (64.5%)		
Yes	31 (29.5%)		38 (35.5%)		
**Monks**	9 (8.6%)		0 (0.0%)		**0.002**
	**Mean**	**SD**	**Mean**	**SD**	***p*-value ^1^**
**Age** (years)	59	6.6	58	6.8	0.177
**DEXA body fatness** (%)	37.7	11.45	36.3	9.48	0.095
**BMI** (kg/m^2^)	28.7	4.12	28.5	4.44	0.748
**Waist circumference** (cm)	96.3	11.82	95.5	11.80	0.587
**Waist-to-hip ratio**	0.95	0.107	0.96	0.112	0.507

^1^ Mann—Whitney test; ^2^ moderate or rigorous; DEXA: Dual-Energy X-ray Absorption; n: sample size; SD: standard deviation.

**Table 2:** Data on vitamins and ferrum levels.

	Fasting Group (n = 105)			Control Group (n = 107)			*p*-Value ^1^
**Variable**	**Mean**	**SD**	**Median**	**Mean**	**SD**	**Median**	
Fe (μg/mL)	99.56	33.4	94	97.37	37	92	0.662
25 (OH) D3 (ng/mL)	17.4	6.3	16.5	17.9	6.7	17.8	0.593
B12 (pg/mL)	330	327	2.2	316	155	0.9	0.681
Folate (ng/mL)	3.9	5.4	272	2.4	4.5	285	0.005

^1^ Mann—Whitney test; Fe: ferrum; n: sample size; SD: standard deviation.

**Table 3:** Summary statistics for mental health variables by group and gender.

	Fasting Group (n = 105)			Control Group (n = 107)			*p*-Value ^1^
**Variables**	**Mean**	**SD**	**Median**	**Mean**	**SD**	**Median**	
GDS	2.24	1.77	2	3.48	2.52	3	**<0.001**
HAS	7.49	4.98	6	9.68	5.24	9	**<0.001**
MMSE	29.15	0.79	29	28.64	1.26	29	**<0.001**
**Males**							
GDS	1.84	1.44	2	3.21	2.20	3	**0.007**
HAS	5.86	3.37	5	7.82	4.25	8	**0.016**
MMSE	29.35	0.68	29	28.94	0.93	29	**0.037**
**Females**							
GDS	2.43	1.90	2	3.63	2.66	3	**0.003**
HAS	8.35	5.48	7	10.56	5.46	9	**0.002**
MMSE	29.04	0.83	29	28.51	1.38	29	**0.003**

^1^ Mann—Whitney test; GDS: Geriatric Depression Scale; HAS: Hamilton Anxiety Scale; MMSE: Mini Mental State Examination; n: sample size.

**Table 4:** Negative binomial regression analysis for GDS scores.

	Model 1 ^1^			Model 2 ^1^			Model 3 ^1^			Model 4 ^1^		
	IRR	95% CI	*p*-Value	IRR	95% CI	*p*-Value	IRR	95% CI	*p*-Value	IRR	95% CI	*p*-Value
**Group**												
Control (ref)	-	-	-	-	-	-	-	-	-	-	-	-
Fasting	0.64	0.52–0.79	**<0.001**	0.67	0.55–0.81	**<0.001**	0.66	0.54–0.81	**<0.001**	0.69	0.56–0.85	**<0.001**
**Sex**												
Males (ref)				-	-	-	-	-	-	-	-	-
Females				1.18	0.96–1.45	0.108	1.14	0.93–1.38	0.206	1.14	0.93–1.39	0.205
**Age (years)**				1.01	1.00–1.03	0.123	1.01	1.00–1.03	0.075	1.01	1.00–1.03	0.089
**Education**												
Primary (ref)				-	-	-	-	-	-	-	-	-
Secondary				0.85	0.63–1.14	0.284	0.91	0.68–1.22	0.528	0.90	0.67–1.20	0.463
Tertiary				0.87	0.63–1.20	0.393	0.95	0.69–1.31	0.767	0.96	0.70–1.31	0.800
Postgraduate				0.63	0.38–1.02	0.061	0.68	0.42–1.09	0.109	0.66	0.41–1.06	0.087
**DEXA body fatness (%)**							1.00	0.99–1.01	0.945	1.00	0.99–1.01	0.986
**Recent weight change**												
No (ref)							-	-	-	-	-	-
Yes							1.49	1.17–1.89	0.001	1.50	1.19–1.89	0.001
**Physical** **activity ^2^**												
No (ref)							-	-	-	-	-	-
Yes							0.83	0.66–1.03	0.092	0.82	0.66–1.02	0.071
**Smoking**												
No (ref)							-	-	-	-	-	-
Yes							1.01	0.76–1.35	0.928	1.01	0.76–1.34	0.961
**Monks**												
No (ref)										-	-	-
Yes										0.54	0.30–0.96	0.034

^1^ Model 1: unadjusted; Model 2: adjusted for sociodemographic parameters; Model 3: further adjusted for body fatness, health status, smoking, and physical activity; Model 4: further adjusted for monk status; ^2^ moderate or rigorous; CI: confidence intervals; DEXA: Dual-Energy X-ray Absorption; GDS: Geriatric Depression Scale; IRR: Incidence Rate Ratio; ref: reference.

**Table 5:** Sensitivity analysis—negative binomial regression analysis restricted to those with a GDS score over the 75th percentile.

	Model 1 ^1^			Model 2 ^1^			Model 3 ^1^			Model 4 ^1^		
	IRR	95% CI	*p*	IRR	95% CI	*p*	IRR	95% CI	*p*	IRR	95% CI	*p*
**Group**												
Control (ref)	-	-	-	-	-	-	-	-	-	-	-	-
Fasting	0.90	0.76–1.06	0.206	0.89	0.75–1.06	0.188	0.92	0.77–1.09	0.333	0.92	0.77–1.10	0.367
**Sex**												
Males (ref)				-	-	-	-	-	-	-	-	-
Females				1.11	0.95–1.31	0.183	1.09	0.95–1.26	0.231	1.09	0.94–1.26	0.269
**Age (years)**				1.00	0.99–1.01	0.731	1.00	0.98–1.01	0.525	1.00	0.98–1.01	0.535
**Education**												
Primary (ref)				-	-	-	-	-	-	-	-	-
Secondary				0.96	0.77–1.21	0.744	0.94	0.75–1.18	0.597	0.94	0.75–1.18	0.600
Tertiary				0.98	0.77–1.24	0.865	0.93	0.73–1.19	0.569	0.94	0.73–1.19	0.590
Postgraduate				0.99	0.68–1.45	0.976	1.01	0.71–1.43	0.945	1.01	0.71–1.43	0.971
**DEXA body fatness (%)**							1.01	1.00–1.01	0.080	1.01	1.00–1.01	0.083
**Recent weight change**												
No (ref)							-	-	-	-	-	-
Yes							1.10	0.92–1.31	0.303	1.10	0.92–1.31	0.308
**Physical** **activity ^2^**												
No (ref)							-	-	-	-	-	-
Yes							1.09	0.92–1.29	0.324	1.08	0.92–1.28	0.345
**Smoking**												
No (ref)							-	-	-	-	-	-
Yes							1.02	0.83–1.25	0.859	1.02	0.83–1.25	0.856
**Monks**												
No (ref)										-	-	-
Yes										0.91	0.75–1.10	0.333

^1^ Model 1: unadjusted; Model 2: adjusted for sociodemographic parameters; Model 3: further adjusted for body fatness, health status, smoking, and physical activity; Model 4: further adjusted for monk status; ^2^ moderate or rigorous; CI: confidence intervals; DEXA: Dual-Energy X-ray Absorption; GDS: Geriatric Depression Scale; IRR: Incidence Rate Ratio; ref: reference.

**Table 6:** Scoring levels of GDS, HAS, and MMSE of the total sample.

	Mean	SD	Median	Skewness
**GDS score**	2.86	2.27	2.00	1.09
0–5 (no symptoms): n = 186 (87.7%)				
6–10 (mild depression): n = 26 (12.3%)				
**HAS score**	8.59	5.23	7.50	1.33
0–17 (mild severity): n = 195 (92.0%)				
18–24 (mild to moderate): n = 13 (6.1%)				
25–30 (moderate to severe): n = 4 (1.9%)				
**MMSE score**	28.90	1.08	29.00	−2.84
<24 (low cognition): n = 2 (0.9%)				

GDS: Geriatric Depression Scale; HAS: Hamilton Anxiety Scale; MMSE: Mini Mental State Examination; n: sample size; SD: standard deviation.

## Data Availability

Data available on request due to privacy and ethical restrictions. The data presented in this study are available on request from the corresponding author. The data are not publicly available due to security.

## Supplementary Materials

The following are available online at https://www.mdpi.com/2072-6643/13/2/627/s1, Table S1: Fasting Periods and Days According to the Rules of the Christian Orthodox Church (A valid license for copyright permission has been obtain from Springer with the number 5006360964783), Table S2: Dietary habits of fasters and non-fasters, Table S3: Frequencies of nutritional intake.
