# Supplementary material for: The Christian Orthodox Church Fasting Diet Is Associated with Lower Levels of Depression and Anxiety and a Better Cognitive Performance in Middle Life
Source: Nutrients. 2021 Feb 15;13(2):627. doi: 10.3390/nu13020627 (PMC7919284; doi:10.3390/nu13020627)
Supplement: Supplementary file 1 [file nutrients-13-00627-s001.pdf]

Table S1. Fasting Periods and Days According to the Rules of the Christian Orthodox Church.

Fasting Period/Days	Duration (d/yr)	Additional dietary restrictions[1]	Fish Consumption
Lent: 6 weeks before the Holy Week of Easter (i.e., Clean Monday to Saturday of Raising of Lazarus)	41	Avoidance of oil and wine on all days except weekends and March 9 (holiday of the Forty Martyrs)	Mandatory on March 25 (Annunciation)
Holy Week: Holy Monday to Holy Saturday	6	Avoidance of oil and wine on all days[2]	
Christmas: November 15 to December 24	40	Avoidance of oil and wine on Wednesdays, Fridays, and Christmas Eve	Mandatory on November 21 (Presentation of Virgin Mary); allowed from November 22 to December 17
Dormition (or Assumption): August 1 to 14	14	Avoidance of oil and wine on all days except weekends	Mandatory on August 6 (Christ's Transfiguration)
Saint Apostles:[3] Monday after All Saints' Sunday Day to June 28	0 to 30[4]	Avoidance of oil and wine on Wednesdays, Fridays, and June 28, unless it falls on a weekend	Mandatory on June 24 (Nativity of John the Baptist)
Important Religious Days: January 5 (Epiphany's Eve), August 29 (Abrupt of John the Baptist's head), September 14 (Exaltation of the Holy Cross)	3	Avoidance of oil and wine, unless they fall on weekends	
Wednesdays and Fridays:[5] All Wednesdays and Fridays except those included in two periods of no food restriction after Easter and Christmas	55 to 63[6]	Avoidance of oil and wine unless they fall on designated holidays	Mandatory on Wednesday of Mid-Pentecost and Wednesday of the sixth week after Easter.
Total	159-197		

[1] In addition to avoidance of foods of animal origin.

[2] Consumption of oil and wine can be tolerated at Holy Thursday's lunch (because oil and wine were consumed at the Last Supper.

[3] So called because it precedes the two apostolic holidays: the feast of Saints Peter and Paul (June 29) and the Council of the Twelve Apostles (June 30).

[4] This varies because its start depends on the mobile feast of Easter. For example, when Easter is celebrated on May 5, the Saint Apostles fasting is not observed.

[5] Strict fasting is preserved as a reminder of the Betrayal (Wednesday) and the Holy Passion of Christ (Friday).

[6] Not counting Wednesdays and Fridays included in all periods described above.

(reproduced with permission from Rodopaios et al 2019 [22])

Table S2. Dietary habits of fasters and non-fasters (controls).

DIETARY HABITS THAT DIFFER BETWEEN THE TWO GROUPS			
**Variable**		**P value (Fisher's exact)**	**GROUP WITH HIGHER CONSUMPTION**
Meat	Beef (140g)	0.000	CONTROLS
	Chicken (140g)	0.011	
	Turkey (30g)	0.002	
	Broth during cooking	0.002	
Eggs		0.034	
Dairy products	Full Fat Cheese (30g)	<0.001	
	Light Cheese (30g)	0.031	
	Milk with chocolate	0.037	
	Yogurt Full Fat (Cow) (10%)	0.001	
	Yogurt Light (Cow) (2%)	0.001	
Sugar	Marmelade	0.008	
	Commercial processed juices	0.014	
	Candies	0.003	
	Refreshments	0.005	
Coffee/Cocoa	Greek, filter, americano, espresso	<0.001	
	Cocoa (1 tea cup.)	0.014	
Alchool	Whiskey, Gin, Vodka, Tequila	<0.001	
	Wine (90ml)	0.018	
	Raki/Ouzo/Tsipouro	<0.001	
Olive oil		0.012	
Seefood	Squid, octopus, fish etc (140g)	<0.001	FASTERS
Tahini		<0.001	
Nuts		0.019	
Fresh seasonal fruits		0.041	
Soya		0.005	
Peanut Butter		0.025	
Halva		0.027	

Table S3. Frequencies of nutritional intake.

CONSUMPTION		FASTERS N=105	CONTROLS N=107	p
**ALCHOOL**	**TYPE OF ALCHOOL**			
	Wine (90ml)			
	Never/rarely	59%	43.9%	**0.018**
	1-3 per month	12.4%	17.8%	
	1-2 per week	22.9%	17.8%	
	3-6 per week	3.8%	11.2%	
	Daily	1.9%	7.5%	
	More than 2 times per day	0%	1.9	
	Beer			
	Never/rarely	71.4%	73.8%	0.915
	1-3 per month	16.2%	14%	
	1-2 per week	10.5%	9.3%	
	3-6 per week	1%	1.9%	
	Daily	0	0.9%	
	More than 2 times per day	1%	0	
	Raki/Ouzo/ Tsipouro (40ml)			
	Never/rarely	80%	51.4%	**<0.001**
	1-3 per month	8.6%	25.2%	
	1-2 per week	7.6%	13.1%	
	3-6 per week	2.9%	1.9%	
	Daily	1%	6.5%	
	More than 2 times per day	0	1.9%	
	Whiskey, Gin, Tequila, Vodka (40ml)			
	Never/rarely	99%	82.2%	**<0.001**
	1-3 per month	1%	14%	
	1-2 per week	0	3.7%	
	3-6 per week	0	0	
	Daily	0	0	
	More than 2 times per day	0	0	
**COFFEE**	**ANY TYPE** (1 tea cup)			
	Never/rarely	39%	19.6%	**<0.001**
	1-3 per month	2.9%	0%	
	1-2 per week	5.7%	1.9%	
	3-6 per week	3.8%	3.7%	
	Daily	21%	20.6%	
	More than 2 times per day	27.6%	54.2%	
	**NUTRITIONAL HABITS**			
**MEAT**	**TYPE OF MEAT**			
	BEEF (140 gr)			**<0.001**

		21%	3.7%	
	Never/rarely	25.7%	34.6%	
	1-3 per month	52.4%	56.1%	
	1-2 per week	1%	4.7%	
	3-6 per week	0	0.9%	
	Daily	0	0	
	More than 2 times per day			
	Chicken (150 gr)			
	Never/rarely	15.2%	7.5%	
	1-3 per month	25.7%	16.8%	**0.011**
	1-2 per week	58.1%	68.2%	
	3-6 per week	1%	7.5%	
	Daily	0	0	
	More than 2 times per day	0	0	
**SEAFOOD**	**SQUID, OCTAPUS, SHRIMS..** (140 gr)			
	Never/rarely	32.4%	59.8%	
	1-3 per month	49.5%	30.8%	
	1-2 per week	18.1%	9.3%	**<0.001**
	3-6 per week	0	0	
	Daily	0	0	
	More than 2 times per day	0	0	
**CHEESE**	**FULL FAT /COW** (1 tea cup)			
		3.8%		
	Never/rarely	1.9%	10.3%	
	1-3 per month	22.9%	1.9%	
	1-2 per week	49.5%	10.3%	
	3-6 per week	12.4%	18.7%	**<0.001**
	Daily	9.5%	36.4%	
	More than 2 times per day		22.4%	
**YOGURT**	**10% FAT /COW** (1 tea cup)			
	Never/rarely	64.8%	66.4%	
	1-3 per month	7.6%	12.1%	
	1-2 per week	19%	4.7%	**0.001**
	3-6 per week	8.6%	11.2%	
	Daily	0	5.6%	
	More than 2 times per day	0	0	
**OLIVE OIL**	(5-6 pieces)			
		10.5%	1.9%	**0.012**
	Never/rarely	1%	0.9%	
	1-3 per month	1.9%	1.9%	

	1-2 per week	8.6%	1.9%	
	3-6 per week	4.8%	7.5%	
	Daily	73.3%	86%	
	More than 2 times per day			
**NUTS**	(5-6 pieces)			
	Never/rarely	21.9%	24.3%	**0.019**
	1-3 per month	17.1%	13.1%	
	1-2 per week	35.2%	21.5%	
	3-6 per week	6.7%	15.9%	
	Daily	14.3%	11.2%	
	More than 2 times per day	4.8%	14%	
**SEASONAL FRESH FRUITS**	(1 piece)			
	Never/rarely	1%	0.9%	**0.041**
	1-3 per month	0%	3.7%	
	1-2 per week	3.8%	11.2%	
	3-6 per week	8.6%	13.1%	
	Daily	35.2%	31.8%	
	More than 2 times per day	51.4%	39.3%	
**TAHINI**	(1 tea cup)			
	Never/rarely	50.5%	80.4%	**<0.001**
	1-3 per month	17.1%	4.7%	
	1-2 per week	21.9%	3.7%	
	3-6 per week	7.8%	0%	
	Daily	1.9%	11.2%	
	More than 2 times per day	1%	0%	
**SOYA**	(1 tea cup)			
	Never/rarely	82.9%	96.3%	**0.005**
	1-3 per month	7.6%	1.9%	
	1-2 per week	9.5%	1.9%	
	3-6 per week	0	0	
	Daily	0	0	
	More than 2 times per day	0	0	
